# Clinicopathological features and treatment paradigms exploration in advanced ampullary cancer

**DOI:** 10.3389/fonc.2026.1934868

**Published:** 2026-09-18

**Authors:** Jiayu Li, Yu Lan, Junliang Chen, Jianying Xu, Yiqin Wang, Lingling Tan, Yize Mao, Ming Luo, Fenghua Wang

**Affiliations:** 1 Department of Medical Oncology, State Key Laboratory of Oncology in South China, Sun Yat-sen University Cancer Center, Guangzhou, China; 2 Department of Medical Oncology, The First People Hospital of Zhaoqing, Zhaoqing, China; 3 Department of Oncology, The First Affiliated Hospital of Jinan University, Guangzhou, China; 4 Department of Medicine II, LMU University Hospital, Munich, Germany; 5 Department of Nephrology, The First Affiliated Hospital, Sun Yat-sen University, Guangzhou, China; 6 Department of Surgery, State Key Laboratory of Oncology in South China, Sun Yat-sen University Cancer Center, Guangzhou, China; 7 Department of Radiation Oncology, Sun Yat-sen Memorial Hospital, Sun Yat-sen University, Guangzhou, China

**Keywords:** advanced/metastatic disease, ampullary cancer, chemotherapy, histopathological subtype, prognosis

## Abstract

**Background:**

Ampullary cancer presents unique clinical challenges due to its rarity and diagnostic complexities. Current data addressing ampullary cancer therapy are limited. This retrospective study focused on advanced disease and explored the rational therapeutic strategies especially based on histopathological subtype, which maybe provide evidence-based guidance for this understudied malignancy.

**Methods:**

We enrolled advanced ampullary cancer patients from two centers. Clinicopathological data and treatment details were collected. Treatment outcome comparisons between different chemotherapeutic regimens (gemcitabine-based vs fluorouracil-based) and different histopathologic types (Pancreaticobiliary vs Non-pancreaticobiliary) were conducted.

**Results:**

71 patients with advanced ampullary cancer were enrolled. Elevated CA19–9 was observed in 63.4% (45/71). Postoperative metastasis occurred in 90.1% (64/71) patients. Histological subtyping was feasible in 43 patients, including 38 pancreaticobiliary, 4 intestinal, and 1 mixed subtype. All patients received first-line chemotherapy with objective response rate (ORR) of 19.7%, median progression-free survival (mPFS) of 9.6 months and median overall survival (mOS) of 33.2 months. Comparable efficacy between gemcitabine-based group (n=27) and fluorouracil-based group (n=44) (ORR 14.8% vs 22.7%, mPFS 6.67 months vs 11.20 months, mOS 26.70 months vs 38.90 months, p>0.05) were observed. Pancreatobiliary subgroup (n=38) exhibited ORR of 15.8%, median PFS of 8.33 months, and OS of 31.7 months, and 20% ORR, 9.93-month PFS, and 32.3-month OS in non-pancreatobiliary cases (n=5).

**Conclusions:**

Advanced ampullary cancer demonstrates aggressive behavior and poor chemotherapy responses. No formal statistical comparison was performed between histopathological subtypes due to the small sample size of the non-pancreatobiliary group. However, the number of patients with the pancreatobiliary-type was far greater than that with the non-pancreatobiliary-type. Due to the underrepresentation of non-pancreatobiliary-type cases, the predictive value of histopathological subtyping in advanced disease could not be adequately assessed.

## 1 Introduction

Ampullary cancer is a relatively rare yet clinically significant entity. accounting for only 0.2% of gastrointestinal malignancies and 7% of all periampullary cancers (1), The surveillance data revealed an annual incidence increase of 0.9% (2).The ampulla of Vater is formed by 3 anatomical components: the ampulla, the intraduodenal portion of the bile duct, and the intraduodenal portion of the pancreatic duct (3).The distinct anatomical complex region of ampullary cancer underlies its histogenetic heterogeneity and histological diversity (4). Although sharing clinical and imaging similarities with periampullary cancers, ampullary cancer requires distinct therapeutic approaches, making its diagnosis and clinical management particularly challenging (5). Ampullary cancer has gained increasing clinical attention in recent years. In March 2022, the National Comprehensive Cancer Network (NCCN) formally classified it as a distinct entity and issued inaugural management guidelines, now updated annually (6).

Ampullary cancer exhibits striking biological aggressiveness and poor prognosis, Nearly half of patients manifest metastases at initial presentation or within 6 months after resection (7, 8). The 5-year overall survival rate of metastatic disease plummets to a mere 4-7% (9). However, current studies on advanced ampullary cancer are small, retrospective, or contain heterogenous populations, which has resulted in limited understanding of clinicopathological features and high-quality evidence-based strategies in clinical decision-making for advanced ampullary cancer. Systemic therapy options may depend on the histologic subtype of ampullary cancer: intestinal type derived from colorectal cancer versus pancreatobiliary/mixed type derived from pancreatic or biliary tract cancer. However, current management protocols largely derive from extrapolated therapeutic frameworks designed for pancreaticobiliary and colorectal malignancies.

Elucidating the clinical characteristics and optimizing therapeutic approaches for ampullary cancer remain imperative, This retrospective study concentrates on advanced-stage disease to characterize its clinicopathological features and optimize its treatment strategies, especially based on histopathological subtype or molecular profile.

## 2 Methods

We identified those patients diagnosed with ampullary cancer at Sun Yat-sen University Cancer Center and Sun Yat-sen Memorial Hospital (January 2000-January 2024). Inclusion criteria include:1)Tumors occured in the center of the ampulla, surround the ampulla, or completely occupied the ampulla, confirming via surgical specimens or imaging. 2) Histologically confirmed adenocarcinoma. 3) Documented advanced unresectable or recurrent/metastatic disease by imaging/intraoperative findings. 4) Received ≥1 first-line chemotherapy regimen (Including patients with *de novo* metastasis and those progressing within 6 months post neoadjuvant/adjuvant therapy.) with baseline imaging evaluation. 5) Availability of complete survival follow-up records. Exclusion criteria include: 1) Concurrent other malignancies. 2) Non-adenocarcinoma histology. 3) Other periampullary cancers (pancreatic/cholangiocarcinoma/duodenal).

Electronic medical records were reviewed to collect data on patients’ demographics, Eastern Cooperative Oncology Group (ECOG) performance status at diagnosis, tumor differentiation grade, histological subtype, metastatic organ, molecular profiles, serum tumor markers, surgical interventions, neoadjuvant/adjuvant therapy utilization, chemotherapy regimens, and efficacy outcomes (objective response rate [ORR], progression-free survival [PFS], overall survival [OS]). Additional pathological parameters included tumor size, histologic grade, and TNM stage (AJCC 8th edition).

Histopathological diagnosis and subtype were confirmed by two independent pathologists in hepatopancreatobiliary multidisciplinary team at Sun Yat-sen University Cancer Center. Based on established criteria, the tumors were classified into one of three histopathological subtypes: intestinal, pancreatobiliary, or mixed (10, 11).Intestinal-type tumors exhibit an immunophenotypic profile similar to colorectal cancers, typically showing positivity for CK20, CDX2, or MUC2 (but negative for MUC1), or co-expression of CK20, CDX2, and MUC2 regardless of MUC1 status (10, 11, 12). Pancreatobiliary tumors exhibit an immunophenotypic profile characterized by MUC1 positivity and CDX2/MUC2 negativity, regardless of CK20 staining, similar to pancreatic/biliary cancers (11, 12, 13).

The choice of first-line chemotherapy regimen (gemcitabine-based vs. fluorouracil-based) was made by the treating oncologists based on a combination of clinical factors, including histological subtype (pancreatobiliary vs. intestinal), Eastern Cooperative Oncology Group performance status, presence of comorbidities, prior exposure to adjuvant chemotherapy, and drug availability at the time of treatment. Treatment decisions followed the National Comprehensive Cancer Network (NCCN) guidelines and institutional protocols, but no strict randomization was applied. Given the retrospective design, regimen selection was subject to physician preference and evolving practice patterns over the long study period (2000–2024), which may introduce confounding by indication.

In the study, ORR is defined as the combined proportion of patients achieving complete response (CR) or partial response (PR). DCR is defined as the combined proportion of patients with CR, PR, or stable disease (SD). First-line mPFS is defined as the time from initiation of first-line chemotherapy until disease progression or death from any cause. First-line mOS is defined as the time from initiation of first-line chemotherapy until death from any cause. Second-line mPFS is defined as the time from initiation of second-line chemotherapy until disease progression or death from any cause. Second-line mOS is defined as the time from initiation of second-line chemotherapy until death from any cause. All assessments followed RECIST 1.1 criteria. Kaplan-Meier method was used to estimate the survival rate,and Log-Lank test was used to analyze the difference between the survival rates from different group patients. Chi-square test and Fisher’s exact test were used to compare the difference in ORR or DCR. Cox proportional hazards model were used to estimate the factors affecting survival rate and calculate the hazard ratios and 95% confidence intervals. To account for potential temporal variations, treatment era (stratified by enrollment year into two periods: 2000–2016 and 2016–2024) was included as a covariate in the multivariate Cox regression model. Clinical information was collected by double-check method, and statistical analysis was performed by R 4.2.0 and Graph Prism 9.5.1.

## 3 Results

From January 1, 2000 to January 1, 2024, 920 patients with ampullary cancers in Sun Yat-sen University Cancer Center and Sun Yat-sen Memorial Hospital were identified. After excluding non-metastatic cases based on inclusion and exclusion criteria, 71 patients with unresectable or recurrent/metastatic disease were enrolled (**Figure 1**).

**Figure 1:**
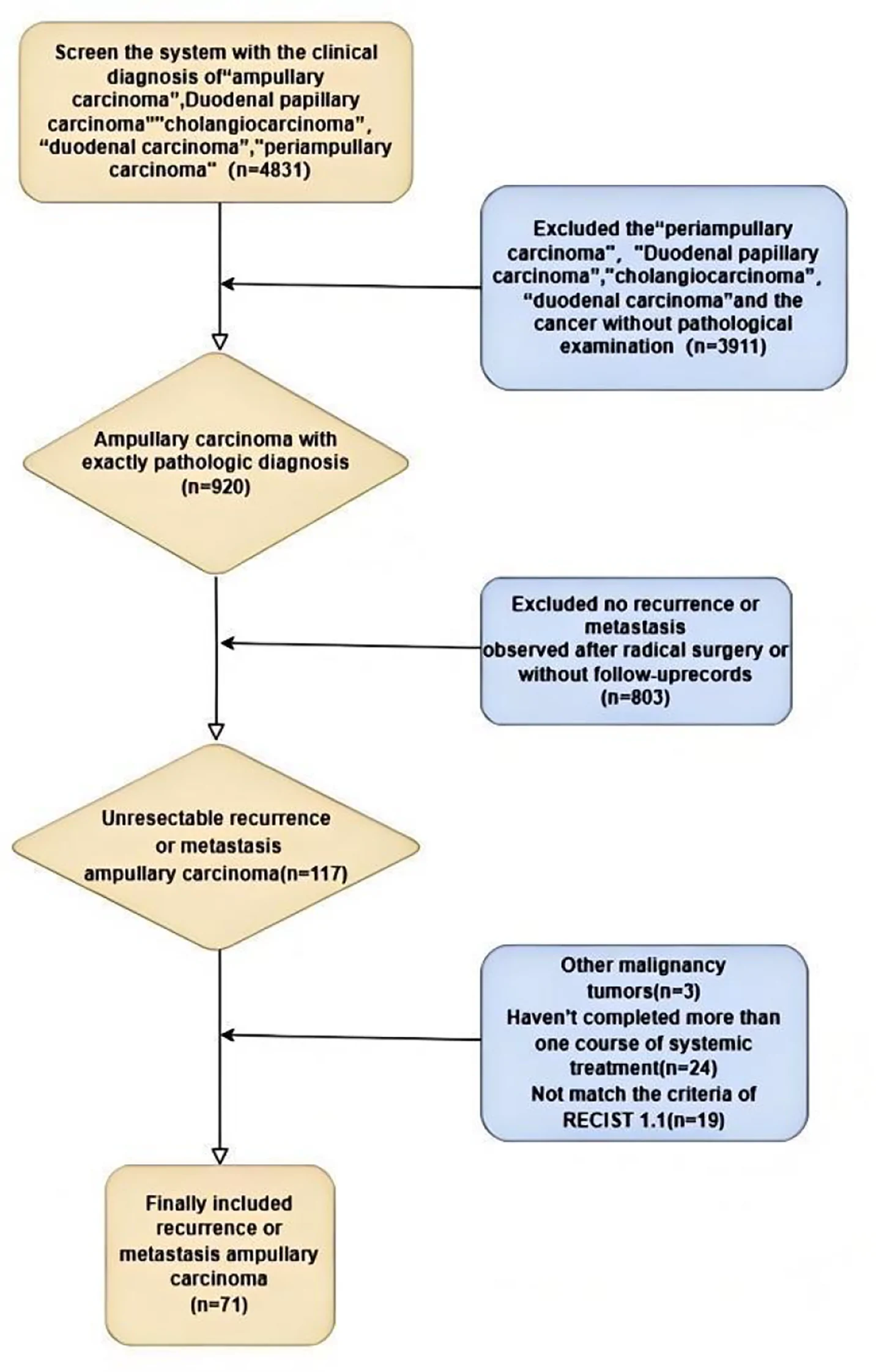
Screening flowchart.

### 3.1 Baseline characteristics

Baseline characteristics are summarized in **Table 1**. The median age was 56.4 years, with male predominance (n=43, 60.6%). The most common symptoms were obstructive jaundice (57.7%) and abdominal pain (35.2%). Metastatic involvement was most common in distant lymph nodes (77.4%), liver (60.6%), and peritoneum (25.4%). Pretreatment CA19–9 elevation (>35 U/mL) occurred in 63.4% of patients Histological subtyping was feasible in 43 patients, including 38 pancreaticobiliary, 4 intestinal, and 1 mixed subtype. Twenty-eight cases remained unclassifiable due to insufficient specimens or missing data. Only 7 patients (9.9%) presented with *de novo* metastasis at initial diagnosis, while the majority (n=64, 90.1%) developed postoperative recurrence or metastasis, with a median time from surgery to progression of 11.6 months (95% CI 8.2-18.2). 23 patients underwent adjuvant chemotherapy after radical surgery. 41 patients in this subgroup who did not receive adjuvant treatment with the following reasons: postoperative complications (n=12), patient refusal (n=10), economic constraints (n=6), lack of adjuvant therapy in early-stage disease (n=5), and loss to follow-up (n=4). All 71 patients received first-line chemotherapy, gemcitabine-based in 27 (38%) and fluorouracil-based in 44 (62%).47 patients (66.7%) proceeded to second-line therapy. Additionally, 20 patients received local interventions (interventional therapy or radiotherapy) alongside systemic treatment.

**Table 1:** Baseline characteristics.

Characteristics	Number(%)
Total	71(100)
Gender	71(100)
Male	43(60.6)
Female	28(39.4)
Age[IQR]	56.44 IQR(51-64)
CA199	71(100)
Elevated (>35)	45(63.4)
Pathological type	43(60.6)
Intestinal	4(5.6)
Pancreatobiliary	38(53.6)
Mixed	1(1.4)
Differentiation	71(100)
Medium-high	44(61.9)
Low	27(38.1)
Metastasis Type	71(100)
Primary metastases	7(9.9)
Postoperative recurrence and metastases	64(90.1)
Adjuvant therapy	23(32.4)
Gemcitabine-based regimen	9(12.7)
fluorouracil-based regimen	10(14.1)
Immunotherapy	4(5.6)
Metastasis Site	
Lymph nodes	55(77.4)
Liver	43(60.6)
Lung	15(21.1)
Peritoneum	18(25.4)
Local therapy	20(28.2)
First-line therapy	71(100)
fluorouracil-based regimen	44(62.0)
Gemcitabine-based regimen	27(38.0)
Second-line therapy	47(66.7)

To evaluate potential selection biases, we compared baseline clinicopathological features for two key treatment decisions: adjuvant chemotherapy (yes vs. no) and first-line regimen (gemcitabine- vs. fluorouracil-based). As summarized in **Supplementary Table S1** and **S2**, both comparisons showed balanced distributions across major prognostic factors—including age, sex, ECOG performance status, tumor differentiation, histological subtype, metastatic sites, N stage, CA19-9 levels, and prior adjuvant therapy (all P > 0.05)—indicating no substantial selection bias in either setting.

Univariate and multivariate Cox regression analyses were performed to identify prognostic factors in advanced ampullary cancer patients. Clinical variables included histological differentiation, histopathological subtype, metastatic sites, serum marker elevation (CEA ≥5 U/ml, CA199 ≥35 U/ml), and time to diagnosis as a covariate. The result identified elevated CA199 (HR = 2.78, 95%CI 1.11-6.90, p=0.029) and peritoneal metastasis (HR = 2.20, 95%CI 1.02-4.80, p=0.046) as independent risk factors.

In the multivariate Cox model, treatment era (2000–2016 vs. 2016–2024) was not significantly associated with survival (P > 0.05). Importantly, the inclusion of treatment era as a covariate did not materially alter the hazard ratios, 95% confidence intervals, or statistical significance of the other independent prognostic factors, suggesting that temporal heterogeneity does not confound our primary conclusions.

The tumor tissues of 21 patients were examined by next-generation sequencing. The most common mutation types were KRAS (61.9%, 13/21), TP53 (33.3%, 7/21) and APC (19.0%, 4/21). In addition,there were 2 cases of BRAF V600E mutation, 2 cases of MSI-H, 1 case of ERBB2 amplification and 1 case of SMAD4 mutation. All patients were TMB-L (median mutation value: 3.45 Muts/Mb)and without germline mutation.(The results are shown in **Figure 2**).

**Figure 2:**
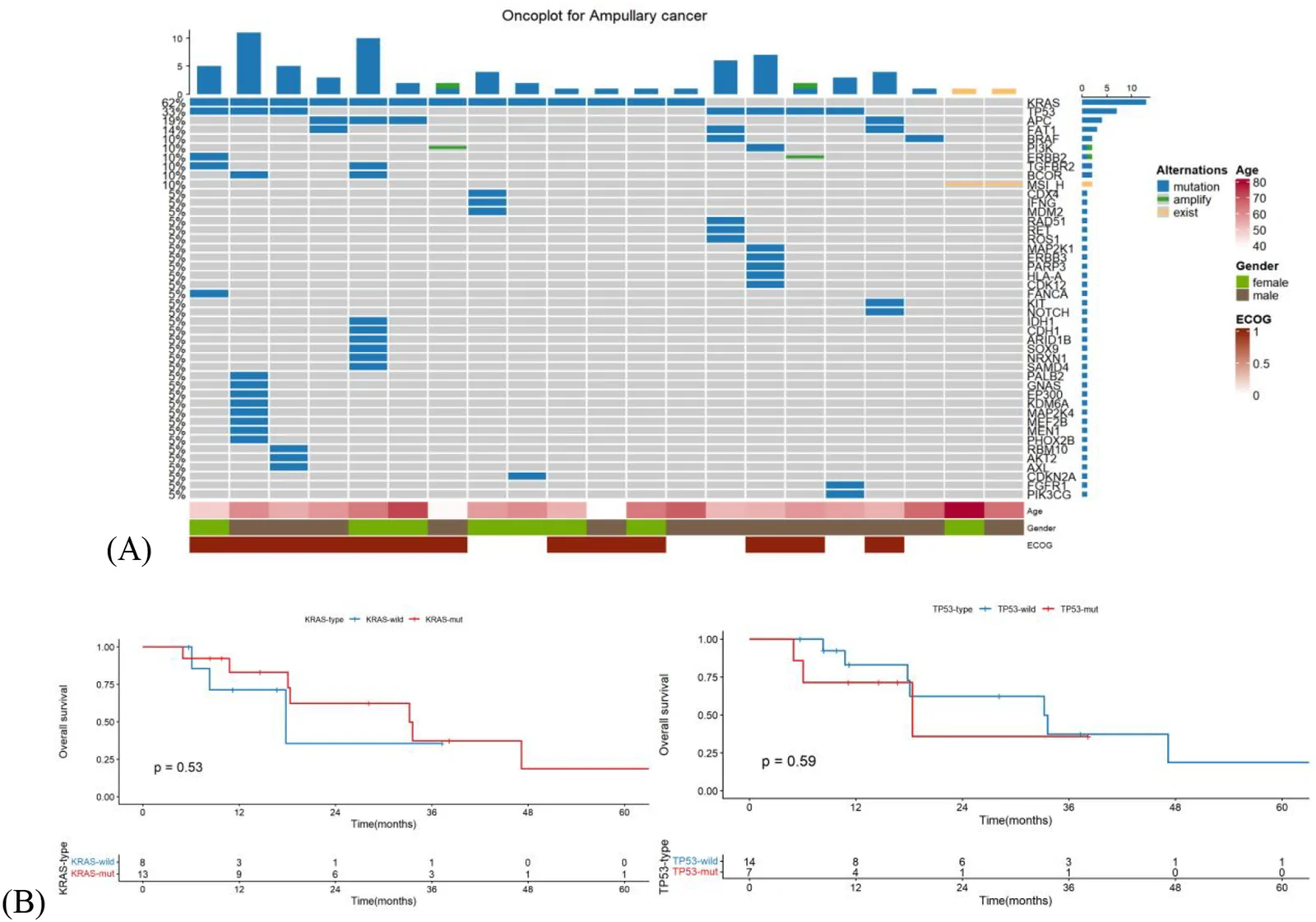
**(A)** The mutation landscape of advanced ampullary cancer. **(B)** Kaplan-Meier survival curves stratified by KRAS and TP53 mutation status.

We subsequently performed survival analyses stratified by KRAS/TP53 mutation status. For KRAS mutation, the hazard ratio (HR) was 1.56 (95% CI [0.37-6.67], P = 0.53), for TP53 mutation, HR was 0.68 (95% CI [0.17-2.76]; P = 0.59). No significant survival differences were detected between wild-type and mutant groups for either gene.

### 3.2 First-line treatment

71 patients received first-line chemotherapy and completed at least one imaging evaluation. 14 (19.7%) achieved PR and 32 (45.1%) had SD, yielding an ORR of 19.7% and DCR of 64.8%. The median mPFS was 9.6 months (95% CI 6.7-13.5 months) and the mOS was 33.2 months (95% CI 26.4-43.0 months).

To assess whether the unexpectedly long median overall survival (33.2 months) was biased by patient selection, we compared survival between patients with *de novo* metastasis (n=7) and those with postoperative recurrence (n=64). The *de-novo* group had a median OS of 38.9 months (95% CI [15.2 - NA]), while the postoperative-recurrence group had a median OS of 33.2 months (95% CI [26.4–45.7]), the difference was not statistically significant (P = 0.63). This analysis suggests that the overall mOS was not driven exclusively by the small *de-novo* subset, but rather reflects the outcome of the predominant recurrent cohort. Sensitivity analysis excluding *de-novo* patients yielded a similar mOS of 33.2 months, supporting the robustness of our primary result.

The results are shown in **Figures 3A, B**. The effect and analysis of first-line treatment are shown in **Table 2**.

**Figure 3:**
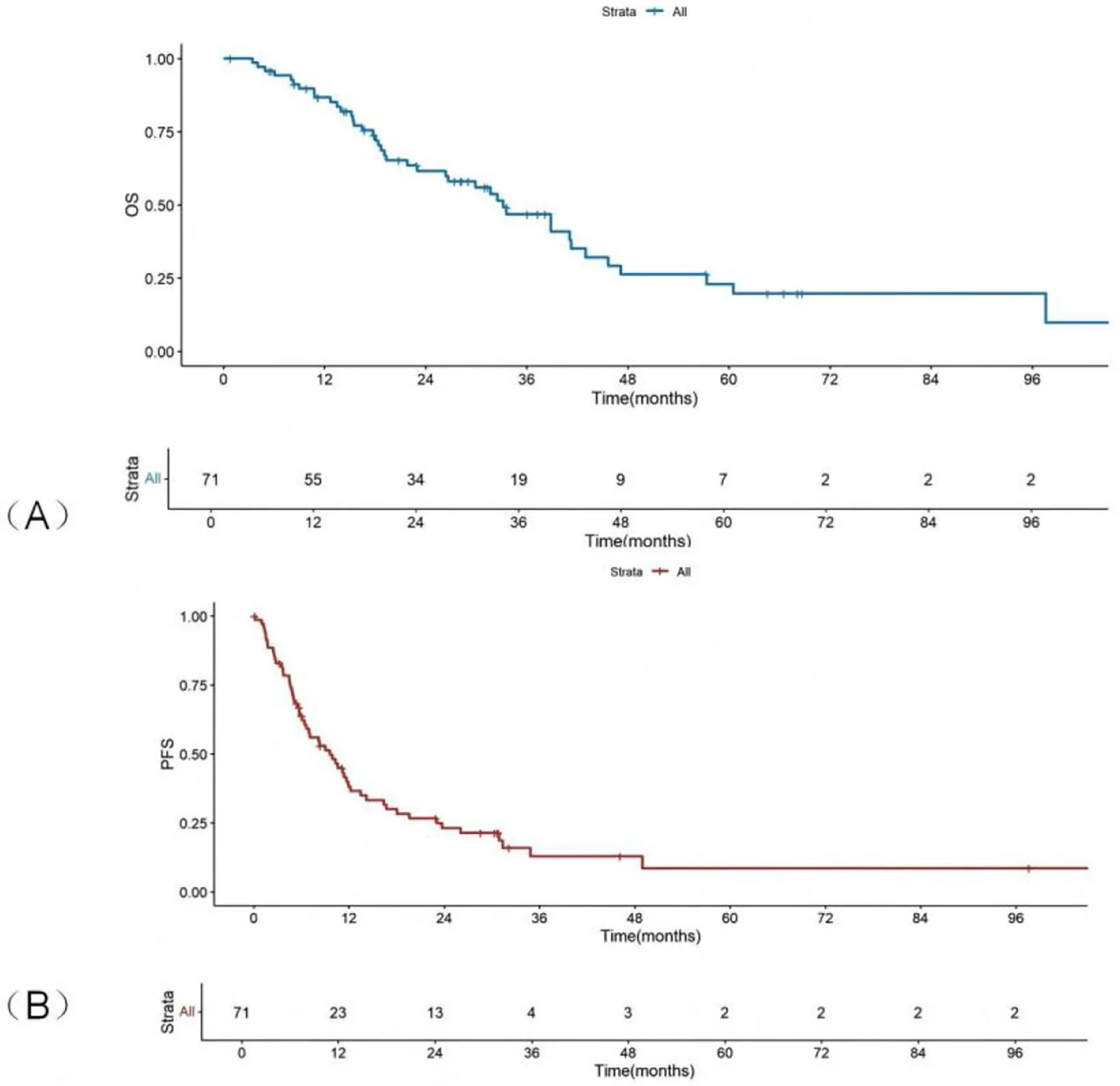
Kaplan-Meier survival curves for advanced ampullary cancer. **(A)** OS and **(B)** PFS.

**Table 2:** Multivariate Cox regression analysis of prognostic factors for overall survival in advanced ampullary cancer.

Variables	Category	HR (95% CI)	P value
Histological differentiation	Low		0.308
	Medium-High	0.69 (0.34-1.40)	
Histopathological subtype	Pancreatobiliary		0.652
	Non-Pancreatobiliary	0.69 (0.14-3.40)	
Serum CEA	Normal		0.670
	Elevated (≥5 U/mL)	1.20 (0.51-2.80)	
Serum CA19-9	Normal		0.029
	Elevated (≥35 U/mL)	2.78 (1.11-6.90)	
Metastatic sites			
Liver	No		0.185
	Yes	1.71 (0.77-3.80)	
Lung	No		0.701
	Yes	1.21 (0.46-3.20)	
Peritoneum	No		0.046
	Yes	2.20 (1.02-4.80)	
Lymph node	No		0.320
	Yes	1.57 (0.64-3.80)	
Treatment era	2000–2016		0.203
	2016–2024	1.83 (0.72-4.70)	

#### 3.2.1 Comparison of different chemotherapeutic regimens (gemcitabine-based regimen vs fluorouracil -based regimen)

Among the study cohort, 27 patients (38%) received gemcitabine-based regimens, including monotherapy with gemcitabine (n = 1), gemcitabine plus cisplatin/oxaliplatin (n = 14), gemcitabine combined with paclitaxel (n = 7), and gemcitabine with capecitabine/S-1 (n = 5). The remaining 44 patients (62%) were treated with fluorouracil-based regimens, comprising S-1/capecitabine monotherapy (n = 11), FOLFIRINOX (n = 9), oxaliplatin combined with S-1/5-fluorouracil/capecitabine (n = 20), and FOLFIRI (n = 2). Comparative analysis revealed an ORR of 14.8% (4/27) in the gemcitabine group versus 22.7% (10/44) in the fluorouracil group (P = 0.61). The mPFS was 6.67 months and 11.20 months for the gemcitabine-based and fluorouracil-based groups, respectively (P = 0.25). Corresponding mOS durations were 26.70 months versus 38.90 months (P = 0.34). The fluorouracil group showed a trend toward improved outcomes without statistical significance.

#### 3.2.2 Comparison of different histopathologic types (pancreaticobiliary vs non-pancreaticobiliary)

Among 38 patients with pancreaticobiliary-type disease receiving first-line chemotherapy, the ORR was 15.8% (6/38), with a mPFS of 8.33 months (95% CI 5.50–16.70) and mOS of 31.70 months (95% CI 23.0–47.20). Subgroup analysis included 21 patients treated with gemcitabine-based regimens and 17 with fluorouracil-based regimens. Comparative outcomes showed ORR values of 14.3% versus 17.6% (P = 0.87), mPFS of 6.67 versus 11.20 months (P = 0.51), and mOS of 29.90 versus 33.20 months (P = 0.24). The fluorouracil-based group exhibited a non-significant trend toward improved outcomes.

For the 5 patients with non-pancreatobiliary-type disease, descriptive efficacy data are as follows: ORR 20.0% (1/5), mPFS 9.93 months (95% CI 5.77–NA), and mOS 32.5 months (95% CI 17.8–NA). No statistical comparisons were performed between the two histological subgroups due to the small sample size of the non-pancreatobiliary group.

### 3.3 Second line and post line treatment

47 patients received second-line therapy after first-line therapy, including chemotherapy alone (n=36),chemotherapy plus anti-angiogenic therapy (n=7), chemotherapy with immunotherapy (n=1) and immunotherapy combined with anti-angiogenic therapy (n=3). The ORR was 8.5% (4/47) and DCR was 57.4% (27/47). The mPFS was 9.57 months and mOS was 20.6 months. Ten patients received anti-angiogenic based regimens,including 7 received FOLFIRI/FOLFOX combined with anti-angiogenic agents (1 PR, 3 SD, 3 PD), while 3 received apatinib plus PD-1 monoclonal antibody (2 SD, 1 PD).

21 patients received local therapy included radiotherapy (n=7), radiofrequency ablation (n=10), hepatic artery perfusion (n=3) and high-intensity focused ultrasound (HIFU) ablation (n=1). The local therapy cohort had a longer mOS (57.4 vs 19.3 months) in an exploratory descriptive comparison; however, this analysis is subject to immortal time bias and selection bias, and therefore should be interpreted as hypothesis-generating only. No formal statistical inference was drawn from this comparison.

## 4 Discussion

Ampullary cancer presents unique clinical challenges due to its rarity and diagnostic complexities. Current data addressing the systemic therapy of ampullary cancer are limited because of no high-quality therapeutic evidence and insufficient consensus in clinical decision-making. This retrospective study focused on advanced ampullary cancer, We collected the clinicopathological characteristics and treatment data and explored the rational therapeutic strategies especially based on histopathological subtype, which maybe provide evidence-based guidance for clinical decision-making of this understudied malignancy.

In this study, *de novo* metastatic ampullary cancer accounted for only 9.8% of cases, with most metastases arising from postoperative recurrence - a pattern consistent with existing literature (4, 11, 14–22).This clinical profile likely reflects the tumor’s distinct anatomical location, where early-stage biliary obstruction symptoms prompt surgical intervention more frequently than in other periampullary cancers (4).Lymph node and liver metastases predominated, while peritoneal and pulmonary metastases demonstrated lower incidence. Notably, peritoneal metastasis emerged as an independent prognostic determinant in advanced disease, mirroring previous observations (13).

However, the recurrence rate stood in marked contrast to the low adjuvant chemotherapy utilization (32.4%), a disparity explained by several clinical realities: *de novo* stage IV disease precluding surgery (9.9%), postoperative complications from pancreaticoduodenectomy delaying or precluding adjuvant therapy, limited pre-2012 evidence for adjuvant chemotherapy in ampullary cancer, and risk-stratified prescribing practices reserving adjuvant therapy for high-risk patients while observing those with early-stage disease.

Systemic chemotherapy remains the cornerstone therapeutic approach for metastatic ampullary cancer with agressive behavior. While emerging evidence (23, 24, 25, 26, 27) has begun mapping the therapeutic landscape through comparative efficacy analyses of various regimens optimal treatment algorithms await further validation in larger cohorts. Retrospective analyses (28, 29, 30, 31, 32) of chemotherpay in advanced ampullary cancer demonstrate modest efficacy, with reported median mPFS of 3–5 months, median OS of 9–12 months, and ORR of around 30.0%. Current regimens for ampullary cancer predominantly consist of gemcitabine-based and 5-fluorouracil-based protocols. While emerging studies have initiated comparative efficacy analyses between these approaches, conclusive evidence regarding differential therapeutic outcomes remains limited. Jun Cheng, Yize Mao et al (14). demonstrated that bioinformatics-based stratification classifies ampullary carcinoma into molecular subtypes with distinct prognostic profiles and differential chemotherapeutic sensitivities. Shoji et al. (29) and ESPAC-3 (15) enrolled non-metastatic ampullary carcinoma cohorts, and compared these different regimens, revealing no statistically significant differences in therapeutic outcomes between the two kinds of approaches. In the study by Dong Kee Jang et al. (16), 255 patients (including 241 with metastatic ampullary carcinoma) received either gemcitabine-cisplatin (GP) or capecitabine-oxaliplatin (CAPOX). Patients treated with GP had a median OS of 20.4 months (95% CI: 17.2–23.6), while those treated with CAPOX had a median OS of 16.0 months (95% CI: 11.2–20.7). It indicated that palliative chemotherapy could improve the survival of advanced ampullary cancer, but did not directly compare the efficacy between the two regimens. Our study enrolled 71 metastatic patients treated with first-line gemcitabine-based (n=27) or fluorouracil-based (n=44) regimens, and the comparative analysis between gemcitabine-based and fluorouracil-based groups demonstrated comparable efficacy across all endpoints (ORR, DCR, mPFS, mOS).

Current ampullary cancer guidelines recommend subtype-driven treatment. Histopathological classification into pancreatobiliary, intestinal, and mixed subtypes retains clinical significance (17, 4). Current evidence demonstrates pancreatobiliary predominance (18), correlating with aggressive tumor behavior characterized by advanced staging, frequent lymph node metastasis, and inferior survival outcomes (11).A retrospective analysis of 170 non-metastatic cases revealed striking survival disparities, median overall survival of 115 vs. 52.5 vs. 94.7 months for intestinal, pancreatobiliary, and mixed subtypes respectively (P<0.001) (19), with pancreatobiliary tumors exhibiting significantly lower 5-year survival rates. However, current management protocols largely derive from extrapolated frameworks for pancreaticobiliary and colorectal malignancies, with conflicting data on the prognostic and predictive value of subtyping. The 2025 NCCN guidelines (20) highlight that existing evidence regarding the independent association between histologic subtypes and adjuvant therapy outcomes remains inconclusive, with some studies demonstrating no significant correlation with treatment response, OS, or disease-free survival (DFS). The landmark multicenter study by Moekotte et al (21) demonstrated survival benefits with gemcitabine-based adjuvant chemotherapy in pancreatobiliary and mixed subtypes, while showing limited efficacy in intestinal-type tumors. While existing data primarily address early-stage disease, our study provides novel insights into metastatic ampullary cancer. Our cohort included 43 subtyped advanced cases (88.4% pancreatobiliary vs. 11.6% non-pancreatobiliary). In our advanced cohort, the pancreaticobiliary subtype (n=38) showed an ORR of 15.8%, mPFS of 8.33 months, and mOS of 31.7 months; the non-pancreatobiliary subtype (n=5) had descriptive values of 20%, 9.93 months, and 32.5 months, respectively. Given the small number of non-pancreatobiliary cases, no comparative statistical analysis was performed, and any observed differences are not interpretable. This discrepancy with prior studies may reflect underrepresentation of non-pancreatobiliary cases in advanced cohorts, suggesting fewer progress to advanced stages-potentially explaining their established better prognosis. Gemcitabine-based (n=25) and fluorouracil-based (n=13) regimens showed comparable efficacy in pancreatobiliary patients.

Previous research on ampullary cancer predominantly focused on chemotherapy, while current investigations have entered the era of immune-targeted therapies. Genetic sequencing serves as a critical tool in identifying patients eligible for targeted therapy and immunotherapy. Genomic profiling in non-metastatic ampullary cancer has established KRAS (30%-40%) and TP53 (20%-25%) as predominant mutations, recognized as early drivers of tumor aggressiveness and predictors of inferior progression-free and overall survival across different histologic subtypes (27–29).Recent advancements (22, 33) in molecular characterization have revealed distinct genomic subtypes of ampullary cancer through next-generation sequencing. However, molecular characterization remains unexplored in advanced disease. Our metastatic cohort exhibited markedly higher KRAS (61.9%) and TP53 (33.3%) mutation rates compared to previously reported early-stage resection series (30-40% and ~20-25%, respectively), suggesting that these mutations may be enriched in advanced disease and potentially drive aggressive progression. Intriguingly, survival analysis demonstrated no significant divergence between KRAS-mutated and wild-type cohorts, a null finding potentially attributable to limited sample size. Existing studies (34, 35) have identified predictive biomarkers for immunotherapy response in ampullary cancer, including PD-L1, Galectin-9, HVEM, IDO, HLA-G, CD8+/FoxP3+ tumor-infiltrating lymphocytes (TILs), and dMMR expression. Targeted therapies remain understudied in this malignancy. In our cohort, 10 patients received anti-angiogenic regimens and 4 patients received immunotherapy, none of which showed exceptional efficacy results, requiring further investigation.

In our exploratory subgroup analysis, patients receiving local therapy (n=20) showed longer median OS compared to those treated with systemic therapy alone (57.4 vs. 19.3 months). However, this finding is subject to substantial biases, including immortal time bias (patients had to survive to receive local therapy) and selection bias (healthier patients were more likely to be referred for local interventions). Given the small sample size and the retrospective nature of this comparison, this observation should be considered hypothesis-generating rather than confirmatory, and warrants prospective validation in larger cohorts.

Our study has limitations inherent to its small-sample retrospective design, and the heterogeneity of treatment regimens further challenges the robustness of our conclusions. Critically, the marked imbalance in subtype representation (pancreatobiliary far outnumbering non-pancreatobiliary) limits our ability to reliably evaluate subtype-guided therapy. Moreover, our study is underpowered to detect differences between subtypes, and the lack of statistical significance should not be misinterpreted as equivalence. We explicitly acknowledge the risk of Type II error. Consequently, our findings are preliminary and insufficient to inform clinical practice, warranting larger prospective studies to determine the therapeutic value of histopathological subtyping in this rare malignancy.

## 5 Conclusion

Advanced ampullary cancer exhibits aggressive behavior with limited chemotherapy efficacy and poor prognosis. First-line treatment with fluorouracil-based or gemcitabine-based combinations showed no statistically significant difference in overall survival, though this finding should be interpreted cautiously given the small sample size. Advanced ampullary cancer is predominantly pancreatobiliary-type histologically, with rare non-pancreatobiliary cases, indicating that histopathological subtyping’s predictive value in advanced disease could not be adequately assessed due to insufficient representation of non-pancreatobiliary cases. The potential role of local therapy and immunotherapy warrants further prospective investigation.

## Data Availability

The original contributions presented in the study are included in the article/**Supplementary Material**. Further inquiries can be directed to the corresponding author.

## Supplementary material

Supplementary Table 1 Baseline Clinicopathological Characteristics of Patients Stratified by Adjuvant Chemotherapy Status

Supplementary Table 2 Baseline Clinicopathological Characteristics of Patients Stratified by Different First-line Chemotherapy Status
