# Supplementary material for: Clinicopathological features and treatment paradigms exploration in advanced ampullary cancer
Source: Front Oncol. 2026 Sep 18;16:1934868. doi: 10.3389/fonc.2026.1934868 (PMC13631782; doi:10.3389/fonc.2026.1934868)
Supplement: Supplementary file 1 [file Table1.pdf]

Table S1 Baseline Clinicopathological Characteristics of Patients Stratified by Adjuvant Chemotherapy Status

Variables	Levels	Without Adjuvant chemotherapy (n = 48)	With Adjuvant chemotherapy (n = 23)	P value
**Age**		56.1 (51.8-63.0)	59.2 (48.0-68.5)	0.26
**Sex**	Male	27 (56.2%)	16 (69.6%)	0.42
	Female	21 (43.8%)	7 (30.4%)	
**ECOG PS**	0	8 (16.7%)	7 (30.4%)	0.12
	1	40 (83.3%)	15 (65.2%)	
	2	0 (0.00%)	1 (4.35%)	
**Histological differentiation**	Medium–high	29 (60.4%)	15 (65.2%)	0.98
	Low	19 (39.6)	8 (34.8)	
**Histopathological subtype**	Pancreatobiliary	25 (52.1%)	13 (56.5%)	0.61
	Non-Pancreatobiliary	5 (10.4%)	0 (0.0%)	
	Unknown	18 (37.5%)	10 (43.5%)	
**N stage**	N0	11 (22.9%)	5 (21.7%)	0.99
	N1–N2	37 (77.1%)	18 (78.3%)	
**Family history**	No	38 (79.2%)	18 (78.3%)	0.99
	Yes	10 (20.8%)	5 (21.7%)	
**Pretreatment CA19-9**	Normal (≤35 U/mL)	14 (29.2%)	12 (52.2%)	0.11
	Elevated (>35 U/mL)	34 (70.8%)	11 (47.8%)	

Table S2 Baseline Clinicopathological Characteristics of Patients Stratified by Different First-line Chemotherapy Status

Variables	Levels	Gemcitabine-based Chemotherapy(n=27)	Fluorouracil-based Chemotherapy (n = 44)	P value
**Age**		54.9 (47.7-62.2)	58.5(51.0-65.0)	0.15
**Sex**	Male	16 (59.3%)	27 (61.4%)	0.99
	Female	11 (40.7%)	17 (38.6%)	
**ECOG PS**	0	5 (18.5%)	10 (22.7%)	0.86
	1	22 (81.5%)	33 (75.0%)	
	2	0 (0.00%)	1 (2.3%)	
**Histological differentiation**	Medium–high	20 (74.1%)	26(59.1%)	0.18
	Low	7 (25.9%)	18(40.9%)	
**Histopathological subtype**	Pancreatobiliary	21 (77.8%)	17 (38.6%)	0.12
	Non-Pancreatobiliary	0 (0.0%)	5 (11.4%)	
	Unknown	6 (22.2%)	22 (50.0%)	
**N stage**	N0	6 (22.2%)	10 (22.7%)	0.99
	N1–N2	21 (77.8%)	34 (77.3%)	
**Family history**	No	18 (66.7%)	38 (86.4%)	0.10
	Yes	9 (33.3%)	6 (13.6%)	
**Pretreatment CA19-9**	Normal (≤35 U/mL)	7 (25.9%)	19 (43.2%)	0.23
	Elevated (>35 U/mL)	20 (74.1%)	25 (56.8%)	
**Adjuvant therapy**	No	21 (77.8%)	27 (61.4%)	0.24
	Yes	6 (22.2%)	17 (38.6%)	
